# A Multiple Synergic Treatment for Non-Healing Ulcer Management in a Patient with *Klippel–Trenaunay* Syndrome

**DOI:** 10.3390/reports6030033

**Published:** 2023-07-20

**Authors:** Cristina Vocca, Gianmarco Marcianò, Vincenzo Rania, Luca Catarisano, Caterina Palleria, Salvatore Ciranni, Giuseppina Torcia, Raffaele Serra, Francesco Monea, Giuseppe Spaziano, Giovambattista De Sarro, Rita Citraro, Luca Gallelli

**Affiliations:** 1 Operative Unit of Pharmacology and Pharmacovigilance, “Renato Dulbecco University Hospital”, Department of Health Science, University Magna Graecia, 88100 Catanzaro, Italy; cristina_vocca@live.it (C.V.); raniavincenzo1@gmail.com (V.R.); lucacatarisano@gmail.com (L.C.); palleria@unicz.it (C.P.); desarro@unicz.it (G.D.S.); citraro@unicz.it (R.C.); gallelli@unicz.it (L.G.); 2 Vascular Surgery Unit, Department of Medical and Surgical Science, University Magna Graecia, 88100 Catanzaro, Italy; s.ciranni@materdominiaou.it (S.C.); g.torcia@materdominiaou.it (G.T.); rserra@unicz.it (R.S.); 3 Research Center FAS@UMG, Department of Health Science, University Magna Graecia, 88100 Catanzaro, Italy; francescomonea@libero.it; 4 Department of Environmental Biological and Pharmaceutical Sciences and Technologies, University of Campania “Luigi Vanvitelli”, 81100 Caserta, Italy; giuseppe.spaziano@unicampania.it; 5 Medifarmagen Srl, Spin Off—University of Catanzaro and Mater Domini Hospital, 88100 Catanzaro, Italy

**Keywords:** Klippel–Trenanauy syndrome, wound, oxygen-ozone therapy, pulsed magnetic fields, nutrients

## Abstract

Klippel–Trenanauy syndrome (KTS) is a rare genetic disease determined by overexpression of the phosphatidylinositol-4-5-bisphosphate 3 kinase catalytic subunit (PIK3CA) gene. The clinical presentation is characterized by venous and capillary malformations and lymphatic malformation. To date, no definitive treatment has been suggested in order to improve the clinical symptoms related to the developments of a skin wound. In this case, we describe a young man with KTS that developed a severe skin wound in the lower right limb unresponsive to the common treatment but responsive to a treatment with oxygen-ozone therapy, pulsed magnetic fields (diamagnetic treatment), and topical fixed association of cocum caprylate, oleic acid, quercetin, and 18-β glycyrrhetinic acid. This is the first case that supports a multistep approach to treat a rare and severe disease, and we hope that other studies can support our data.

## 1. Introduction

Klippel–Trenanauy syndrome (KTS) is a rare genetic syndrome manifesting in infancy and progressing in adolescence and adulthood [1]. It is estimated that there are two to three cases of KTS per 100,000 live births without sex or ethnic predilection [2].

According to the International Society for the Study of Vascular Anomalies 2018, KTS consists of capillary and venous malformations, limb overgrowth, and lymphatic malformations [3]. 

KTS is linked to mutations in the phosphatidylinositol-4-5-bisphosphate 3 kinase, catalytic subunit (PIK3CA) gene, responsible for the growth of cells and the development of tissues in the body, through modifications in mTORC2 pathways [4]. 

Persistent embryonic veins, (i.e., lateral margin vein and/or persistent sciatic vein), may lead to varicosities, pain and skin ulcers, thrombophlebitis, and thromboembolism [5]. Moreover, venous malformations in the pelvis and abdominal organs can cause internal bleeding [5]. 

Musculoskeletal alterations can cause pain, limb enlargement (e.g., dysmetria), and movement disorders. Lymphatic malformations are present in 15–50% of the patients. Developmental delay, seizures, and genitourinary alterations are rarer clinical findings [1]. 

Echo-color Doppler ultrasound and magnetic resonance imaging (MRI) are used to characterize the entity of vascular malformations and soft tissue/bone hypertrophy [6].

In 1907, Frederick Parkes Weber described two patients with limb enlargement, port-wine stains, and arteriovenous malformations of the affected limb with detectable pulsation and thrill [7]. 

The presence of arteriovenous malformation is the criterion for distinguishing Parkes Weber syndrome from Klippel–Trenaunay syndrome [8]. In fact, both Parkes Weber and Klippel–Trenaunay syndrome have capillary malformation (CM), venous malformation (VM), and lymphatic malformation (LM); however, only Parkes Weber syndrome has arteriovenous malformations, which is relevant for treatment strategy [8]. 

In both patients, the skin wound represents a clinical problem due to the longtime of healing and a low efficacy of local treatment [9]. 

In patients with skin wounds, a treatment with tissue debridement, topical antimicrobial, and compression was proposed [10,11,12]; however, a non-pharmacological treatment with diamagnetic therapy can also be used [13] due to its anti-inflammatory, anti-oedema, and neuroprotective effect [14,15]. Another opportunity for skin wound treatment is oxygen-ozone therapy. It exerts anti-inflammatory and antioxidant actions through the downregulation of prostaglandins bradykinin and reactive oxygen species, and the upregulation of both nitric oxide and nuclear factor erythroid 2–related factor 2 (Nrf2) pathways [16]. 

In previous clinical studies, we reported that pulsed electromagnetic field [13,17], as well as topical regenerative nutrients [18], are useful in in patients with skin wounds.

To date, no study has described a definitive non-surgical treatment of skin wounds in patients with KT syndrome. In fact, drug treatment does not always have efficacy, other treatments are not studied, while surgery in the treatment of varicosities and venous malformations is controversial because it can be complicated by infection, lymphorrhoea, and skin necrosis [19]. Some surgeons feel that surgery only provides a temporary improvement and may damage venous and lymphatic structures, leading to increased oedema in the affected limb. Bergan et al. [20] treated the skin wounds of nine patients with KT syndrome with ultrasound guided foam sclerotherapy failing to report a definitive clinical effect. 

In this study, we describe a young man with KT syndrome that developed a skin wound unresponsive to several treatments, that was successfully treated with a pharmacological and non-pharmacological topical treatment. 

## 2. Detailed Case Description

A 22-year-old man affected by KT syndrome presented to our ambulatory of pain medicine for a refractory lower right limb pain ulcer. 

Previous studies have documented the presence of the typical sign and symptoms of KT syndrome (Table 1). Recent history revealed the presence of intracranial (right side) angioma and seizures and glaucoma in treatment with phenobarbital (150 mg/day) and latanoprost/timolol collyrium, respectively. 

**Figure 1:**
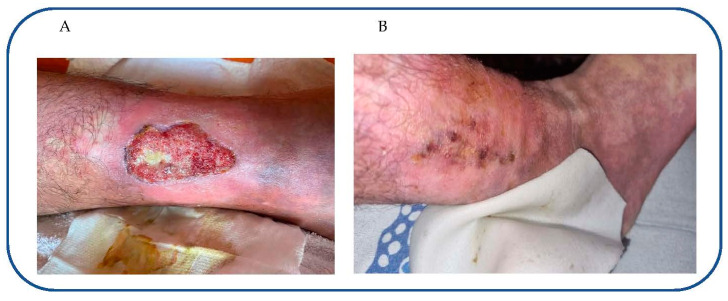
Skin wound at the time of admission (**A**) and at the end of the treatment (**B**).

At the admission, clinical evaluation showed that the patient was temporo-spatial oriented, with a hemangiomas and port-wine discoloration in the right side of the body (from scalp to lower limb). Blood pressure was 120/68 mmHg with a heart rate of 65 b/min; body temperature was 36.2 °C, and body mass index was 28.2. The evaluation of abdominal and respiratory function excluded the presence of other diseases. The patient denied the use of smoke, licit or illicit drugs, or trauma to the extremity. He had been managed at an outpatient wound care facility with local wound care and compressive therapy. The patient’s vascular examination demonstrated a sore skin wound (length 6 cm, width 3 cm) along the gaiter distribution of his right lower extremity more consistent with venous stasis disease than an infectious etiology. Wound edges evaluation revealed a thin and frail skin that stretches after topical debridement. The patient and his father referred that he used several topical treatments (josamycin and other topical antimicrobial drugs, lidocaine, cadexomer iodine gel, diflucortolone and other topical corticosteroids, collagenase, and hyaluronic acid) without clinical improvement. Considering the type of disease (genetic disease), its epidemiology (rare disease), the previous treatments, the persistence of local pain (visual analogical scale, VAS: 8), and dermal wound (Figure 1A), we started a topical treatment bis in week with oxygen-ozone (10 mcg) directly injected along the borders of the ulcer (time of treatment 5 min). In agreement with our previous study, the gas mixture was obtained using an ozone generator (Ozo2 Alnitec, Cremosano (CR), Italy) connected to a pure O2 source [16]. After the injection, the skin changed the colors from red to white, a compression bandage was applied on the wound. Three days later, at the time of the second treatment with oxygen-ozone, the bandage was removed, but due to the skin frailty, the edges of the wound remained attached to the bandage with an increase in wound width (from 3 cm to 3.2 cm). Clinical evaluation documented a little improvement of pain during the days after the first treatment (VAS 7.5), so we continued the treatment. After oxygen-ozone administration we used a compression bandage containing ozonized water. During subsequent administrations, we did not observe lesions at the borders during the remotion of bandage, and we recorded a decrease in pain (VAS 7) without a reduction in wound size (Table 2). Therefore, we started a topical treatment with diamagnetic therapy (CTU MEGA 20 Diamagnetic Pump MOD07-2-5; frequency of 5 HZ, with magnetic flux density of 86 MT; protocol: movement of liquids: Intra L-Extra H for 10 min; Endogenous bio stimulation: cellular membrane for 15 min) after the topical administration of oxygen-ozone. During each session of treatment (time of treatment 30 min), the patient was in a sitting position with the lower leg and foot supported with a foot stand. After four sessions of treatment (every three days), we recorded a significant improvement of pain (VAS: 4), with a moderate improvement of wound size (4.8 cm × 2.3 cm). Therefore, we added to this protocol a topical treatment with Ferialt^®^ cream (a fixed combination of coco-caprylate, oleic acid, quercetin and 18-β glycyrrhetinic acid) on the ulcer surface and then applied the bandage with a gauze soaked in an ozonated solution. This protocol was used for about two months (14 sessions of treatment, 2 sessions every week) when we recorded a complete improvement of the wound (Table 2).

## 3. Discussion

We described the management of the wound on the lower right limb using a pharmacological and non-pharmacological approach in a young man with Klippel–Trenaunay syndrome.

KT syndrome is clinically characterized by a triple-featured condition in which small thread-like capillaries (hemangiomas and port-wine discoloration) are malformed, bone and soft-tissue structures are hypertrophied, and the venous system is malformed causing varicosities [1].

It requires a complex and multi-professional management; symptomatic treatment represents the main strategy to both correct the macroscopical defects and resolve/prevent acute episodes [21].

Hemangiomatosis is the most common cutaneous manifestation of KTS and is usually present from birth [9].

In the present case, a 22-year-old man with Klippel–Trenaunay syndrome, epilepsy, and an history of comorbidity surgically treated was presented to our observation for the development of a skin ulcer in the lower right limb.

Previously, it has been documented that in patients with venous insufficiency, skin care prevents infection and bleeding, while the orthopedic examination establishes any limb discrepancies and the need for surgery [1].

In the present case, we used a multimodal approach with oxygen-ozone therapy, diamagnetic therapy, and topical cream.

Oxygen-ozone therapy has controversial benefits in wound management related to device employed, method of administration, concentration used, and ability of physician.

Oxygen-ozone acts through multiple antioxidant and anti-inflammatory properties stimulating bacterial cell apoptosis. Furthermore, red blood cells become more flexible, increasing oxygen arrival to tissues, and accelerating wound healing. The analgesic effect may be obtained through the activation of opioid system, and the expression of antioxidant and chemotactic factors, and the reduction in pro-inflammatory cytokines [22].

Several clinical studies documented the efficacy and the safety of oxygen-ozone therapy in wound healing in diabetic patients with skin wounds [23,24,25].

Wainstein et al. [23], evaluated the effect of a 12-week treatment with oxygen-ozone documented its efficacy in wound healing through a reduction in blood viscosity, and both bactericidal and anti-edema property.

Liu et al. [24], analyzing data from 212 participants with diabetic wounds, documented the efficacy of oxygen-ozone versus antibiotics in reducing wound size and risk of hospitalization, but not in increasing the number of wounds healed.

Fitzpatrick et al. [25], reviewing nine clinical trials (four randomized clinical trials), highlighted the efficacy and the safety of oxygen-ozone therapy in wounds healing.

More recently, in 114 patients with venous leg ulcers, Pasek et al. [22] compared the effects of topical hyperbaric oxygen versus topical oxygen-ozone and documented that both treatments improved clinical symptoms.

In agreement with these studies, our patient oxygen-ozone therapy reduced the pain intensity, probably through the effects on vessel tissue, but did not improve the wound size.

The use of diamagnetic therapy, also named as pulsed magnetic fields (PEMFs), significantly reduced the wound size and reduced the pain intensity. PEMFs are a versatile physical therapy based on the application of low-frequency, high-intensity pulsed magnetic fields able to induce anti-inflammatory, anti-oedema, and neuro-modulatory effects. Anti-inflammatory effects are related to the up-regulation of adenosine A2A and A3 receptors [26], the decrease in cytokines release [27] and the inhibition of NF-κB [28,29]. The anti-oedema effect is related to diamagnetic effects, moving fluids according to ion movement [30]. However, the decrease in inflammatory cytokines as well as the pro-angiogenic effect contribute to the anti-edema effects [31]. The neuro-modulatory activity is mainly related to ion channels modulation (especially sodium, potassium, and calcium channels) [32,33,34], but also to a neuroprotective effect associated with the brain-derived neurotrophic factor (BDNF) increase [35].

Despite the presence of preclinical evidence of PEMFs efficacy in wound management [36], few clinical trials have been conducted.

In patients with venous ulcer, Ieran et al. [37] reported that a 90-day treatment with PEMFs improved clinical symptoms without ulcer worsening.

Stiller et al. [38] proposed a PEMFs portable device for the management of venous ulcers. The device was applied 3 h daily for 8 weeks. Significant improvement was observed at week 4 and 8 in pain perception, increase in granulation tissue, wound area, and depth. No side effects were reported.

Kwan et al. [39] studied 13 patients with diabetic wounds treated for 3 weeks with PEMF (7 patients) or placebo (6 patients). PEMF reduced wound size (18% vs. 10%) and increased the cutaneous capillary blood velocity (28%) and the capillary diameter (14%).

In a patient with a mixed foot ulcer, we recently documented the clinical efficacy and safety of PEMF in improving both wound size and pain perception [13].

In the present case, the treatment with both oxygen-ozone and PEMF reduced pain perception, probably through a synergistic effect related to anti-inflammatory and antinociceptive activity but failed to improve the wound size.

Therefore, to improve skin care by reducing the development of infections, we applied a topical treatment with Ferialt^®^, a fixed combination of coco-caprylate, oleic acid, quercetin, and 18-β glycyrrhetinic acid.

Rodrigues et al. [40], in an experimental animal model, showed that oral oleic acid favors wound healing, reducing inflammatory cytokines.

In an experimental model, we showed that quercetin and oleic acid (specifically an ester derivative) improved skin lesions through the activation of G protein-coupled receptor 40 (GPR40) that induces TGF-β production and MMP-9 release [18]. Oleic acid stimulates immune response in the ulcer healing process, restoring the inflammatory acute phase; quercetin inhibits the pathway of cyclooxygenases and phosphatidylinositol 3 kinase, inducing antiedematous, antiplatelets, and anti-inflammatory effects.

In agreement with this study, we documented in 56 patients with diabetic foot wounds, that the topical administration of quercetin plus oleic acid in nano-hydrogel formulation reduces both wound healing time and pain intensity without the development of adverse events [41].

18-β glycyrrhetinic acid, the major active component of licorice root extract, has anti-inflammatory effects (through the inhibition of pro-inflammatory genes and cytokines, of nitric oxide synthase) and antimicrobial effects [42]. Hung et al. [43] reported an increased proliferation of human dermal fibroblasts and HaCaT keratinocytes after the incubation with 18-β glycyrrhetinic acid. The active substance increased aquaporin-3 expression, and the phosphorylation of protein kinase B (Akt), extracellular signal-regulated kinase (ERK), and p38 suggest a role in wound healing. In an experimental model, Hara et al. [44] reported that cutaneous wound healing was impaired in knockout mice for acquaporin-3.

In conclusion, in the present case report, we suggest that oxygen-ozone therapy, diamagnetic therapy, and local Ferialt^®^ have a synergistic mechanism capable of reducing the intensity of pain and inducing complete healing of the wound without the development of adverse drug reactions.

This case presents some limitations related to the type of study and the impossibility of carrying out an experimental study able to explain the mechanism of the synergy. KTS is a rare genetic syndrome with no specific treatment and wounding is a serious clinical problem in these patients. If there are no other clinical data, the treatment is only symptomatic. The treatment suggested in the present study may be beneficial in some patients. However, the vulnerability of patients requires close follow-up to monitor for any recurrence.

## Figures and Tables

**Table 1:** Previous history of enrolled patient.

Year	Activity
2002	Surgery for the correction of hypospadias and hydrocele
2004	Abdominal ultrasound evidenced renal asymmetry (right kidney major than left, longitudinal diameter 9.4 vs. 7.7 cm)
2014	Asymmetry in lower limbs length (right longer than left) with a pelvic misalignment
2015	Orthopedic surgery to correct hypermetria using a left tibial lengthening and right femoral epiphysiodesis
2015	Doppler ultrasound highlighted ectasia, tortuosity, and medialization of right saphenous vein
2016	Diagnosis of refractory epilepsy
2016	Magnetic resonance angiography imaging: venous flow alteration in right cerebellum hemisphere and cortical calcification in right occipital and temporoparietal lobes
2018	Ultrasound color Doppler: congenital malformation of vascular system: deep left and superficial right inferior limb circulation were not in communication, while the superficial circulations were linked
2021	Increase in γ-GT serum levels (70 U/L, normal range 5–40) and reduced D vitamin serum levels (14 ng/mL; normal range > 30)

**Table 2:** Effect of the treatment of skin wound in the enrolled patient during each session of treatment (1 admission, 18 end of the treatment) on both pain and dimensions. VAS: Visual analogical scale; O-O: Oxygen-ozone; DM: diamagnetic therapy; Ferialt^®^: cream, fixed combination of coco-caprylate, oleic acid, quercetin and 18-β glycyrrhetinic acid.

Session of Treatment	VAS	Wound Dimension (cm)	Treatment	Time of Treatment (Minutes)
1	8	6 × 3	O-O	5
2	7.5	6 × 3.2	O-O	5
3	7.0	6 × 3	O-O	5
4	6	5.4 × 2.7	O-O, DT	30
5	5.5	5.2 × 2.5	O-O, DT	30
6	5	5.0 × 2.4	O-O, DT	30
7	4	4.8 × 2.3	O-O, DT	30
8	4	4.5 × 2.2	O-O, DT, Ferialt^®^	35
9	3	4.2 × 2.0	O-O, DT, Ferialt^®^	35
10	3	3.5 × 1.8	O-O, DT, Ferialt^®^	35
11	3	3.1 × 1.5	O-O, DT, Ferialt^®^	35
12	2	2.5 × 1.2	O-O, DT, Ferialt^®^	35
13	2	2.1 × 0.9	O-O, DT, Ferialt^®^	35
14	1	1.5 × 0.7	O-O, DT, Ferialt^®^	35
15	1	1.3 × 0.5	O-O, DT, Ferialt^®^	35
16	0	0.9 × 0.4	O-O, DT, Ferialt^®^	35
17	0	0.6 × 0.3	O-O, DT, Ferialt^®^	35
18	0	0.3 × 0.2	O-O, DT, Ferialt^®^	35
19	0	0.2 × 0.1	O-O, DT, Ferialt^®^	35
20	0	0.1 × 0	O-O, DT, Ferialt^®^	35
21	0	0	O-O, DT, Ferialt^®^	35

## Data Availability

All data generated during this study are included in this article. Further enquiries can be directed to the corresponding author. The data are not publicly available due to privacy.
